# Cognitive distortion based explainable depression detection and analysis technologies for the adolescent internet users on social media

**DOI:** 10.3389/fpubh.2022.1045777

**Published:** 2023-01-17

**Authors:** Bichen Wang, Yanyan Zhao, Xin Lu, Bing Qin

**Affiliations:** Social Computing and Information Retrieval Research Center, Harbin Institute of Technology, Harbin, China

**Keywords:** depression detection, cognitive distortion, cognitive psychology, social media, data mining, adolescent psychology

## Abstract

Nowadays, adolescents would like to share their daily lives via social media platforms, which presents an excellent opportunity for us to leverage these data to develop techniques to measure their mental health status, such as depression. Previous researches focus on the more accurate detection of depression through statistical learning and ignore psychological understanding of depression. However, psychologists have given lots of theoretical evidence for depression. Such as according to cognitive psychology research, cognitive distortions will result in depression. Thus, in this study, we propose a new task, explainable depression detection, to not only automatically detect depression but also try to give clues to depression based on cognitive distortion theory. For this purpose, we construct a multi-task learning model based on a pre-trained model to detect depression and identify cognitive distortion. And we use many analytical means including word clouds for data analysis to draw our conclusion. Previous social media users' depression corpus and our cognitive distortion corpus are utilized for analysis and experiment. Our experimental results outperform the baseline results and interesting conclusions about adolescent depression are drawn.

## 1. Introduction

At present, people's mental health problems have become more and more serious. During the COVID-19 pandemic, 53.8% of Chinese residents rate the psychological impact of the COVID-19 outbreak at moderate to severe levels, including 16.5% with depressive symptoms and 28.8% with anxiety (1). The psychological impact of the crown pneumonia epidemic on adolescents is more serious, 22.28% of adolescents are suffering from depressive symptoms in China (2). Among these psychological problems, depression, or called Major Depressive Disorder (MDD) is the most common one. Previous research has proved the language used by people with depression is different, such as more focus on self and detachment from the community, which prompts us to use language to automatically detect depression (3).

A large number of people, especially adolescents, are more willing to share their information on social media and people with depression also post their life events without directly interrupting or disturbing someone and get emotional support from it (4). These social media posts constitute a user's electronic footprint on the internet, which contains information about the user's daily life. Therefore, social media posts are useful to detect latent depressive risk.

With the development of statistical learning techniques, a large number of statistical learning methods have been applied to develop automatic depression detection tasks, such as BoW (Bag of words) and SVM(Support Vector Machine) (5, 6). Classic deep learning methods such as convolutional neural networks (CNN) and recurrent neural networks (RNN) have demonstrated their superiority in depression detection (7). Recently, the pre-trained model such as BERT has been introduced to improve the performance (8). However, most of the current researchers just focus on predicting whether the user is depressed or not based on linguistics statistical characteristics. They try to model the data using various statistical learning methods but ignore the long-running psychological research on depression. **On the one hand**, they do not take the psychological understanding of depression into account. **On the other hand**, simply giving a binary judgment of depression will not help professionals in subsequent treatment and intervention. In our research, we provide psychologists with effective clues about depression according to psychological theory, which can not only increase the credibility of the model but also guide subsequent psychologists' treatment.

In our research, the theory of cognitive distortion about depression is introduced to depression detection. Although the root of depression is still unclear in the field of psychology, psychologists have conducted extensive research and discussions on the occurrence and treatment of depression. Beck's cognitive distortion theory of depression believes that depression is caused by cognitive distortion (9, 10). A cognitive distortion is defined as a person's inaccurate perception of the real world, and it can reinforce negative thoughts and lead to depression. As shown in Table 1, these are common cognitive distortions, which represent people's inaccurate perceptions of the world. Cognitive distortion theory research on depression has provided us with a new perspective that we can assist detection of depression by capturing cognitive distortion languages. These cognitive distortion languages can be computed by computer, which has been proved by some word-level features in various aspects (11). We will go further by proposing the cognitive distortion classification task to automatically identify cognitively distorted sentences using existing natural language processing techniques.

**Table 1:** These are examples of cognitive distortions.

**Catagory**	**Example**
Disqualifying the positive	I got good grades on the test, but that was just too easy this time and I got lucky.
Mindreading	He was looking at me just now, and he must think I'm ugly.
Fortune-telling	I'm sure something bad will happen to me tomorrow.

They are people's incorrect perceptions of the world.

Introducing cognitive distortion theory can also provide model explanations. As mentioned above, cognitive distortion theory provides a potential possible clue for depression. If we identify the cognitive distortion language of depressed patients, we can allow professionals to understand and treat depression via cognitive distortion theory. It also makes it easier for psychologists to correct cognitive distortions. This is more valuable than simply giving a binary judgment of depression.

**Figure 1:**
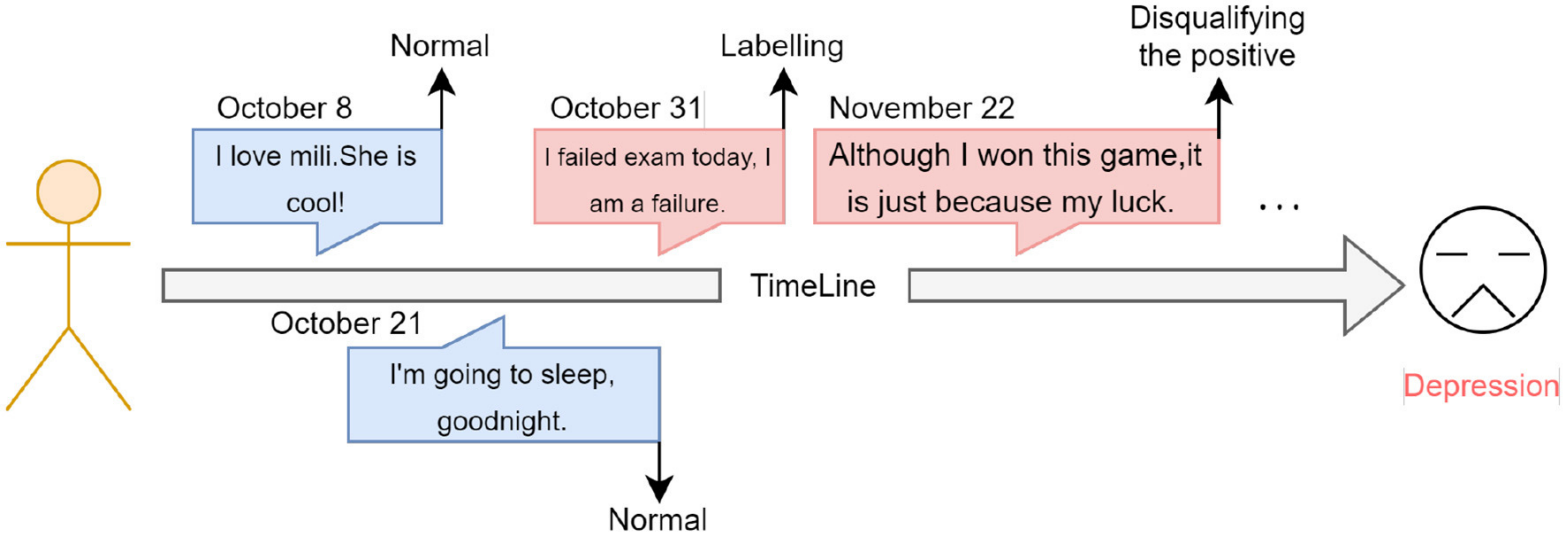
Our task is shown in the figure, labeling cognitive distortions at the post-level and depression at the user-level. The cognitive distortions are labeled with the 11 cognitive distortions.

Because cognitive distortions are very associated with depression, we analyze and exploit this relationship from the perspective of deep learning techniques. As Figure 1 shows, by combining depression detection with cognitive distortion detection, we construct an explainable depression detection model. The model has gotten the detection ability for cognitive distortions and improved the ability to detect depression. It is also better to help professionals treat depression by presenting cognitive distortion information. We use an advanced language model, BiDirectional Encoder Representation From Transformers (BERT) (12) to identify cognitive distortion language in social media posts of users and aggregate this information to obtain user representations to guide the depression detection model. Finally, our model can detect post-level cognitive distortion information and user-level depression. In this way, our model can not only detect depressed users more convincingly but can also help further correct this distorted information.

In our study, we establish systems that could perform both tasks simultaneously, detecting depression and cognitive distortions. At the same time, we use many means to analyze the results obtained by the model, especially the cognitive distortion phenomenon in adolescents with depression, and we draw some interesting conclusions. In our research, we also create a cognitive distortion dataset and extend it to the social media domain using a semi-supervised approach. Experimental results show that the system outperforms the baseline model. Our research takes a step toward combining artificial intelligence technology with cognitive psychology, providing a new perspective for developing psychology-related artificial intelligence.

Our contributions are:

1. As far as we know, we are the first to use deep learning techniques to detect depression based on cognitive distortion. We explore the feasibility of introducing psychological conclusions into natural language processing.
2. We have examined the relationship between cognitive distortions and adolescent depression in psychology using deep learning techniques. At the same time, we also analyzed the differences in cognitive distortion in patients with depression in different age groups.
3. We construct a small publicly available corpus of cognitive distortions. At the same time, we extend it to social media data. For the first time, we combine the two tasks to improve the performance and interpretation of depression detection.

## 2. Cognitive distortion based explainable depression detection method

In this section, we introduce our cognitive distortion based explainable depression detection method. We first introduce the cognitive distortion theory and the relationship between cognitive distortion and depression. We then introduce a cognitive distortion classification task. Finally, we propose a new technique to detect both cognitive distortions and depression automatically.

### 2.1. Cognitive distortion theory on depression

#### 2.1.1. Definition of cognitive distortion and relationship to depression

Aaron Temkin Beck developed the Cognitive Distortion Theory in his research on depression (10, 13). And on this basis, he put forward the treatment of depression, Cognitive Behavioral Therapy (CBT). Cognitive distortions are thoughts that cause an individual to perceive reality improperly. Such improper information processing affects the patient's mood, behavior, and physiology so that the patient's social and interpersonal functioning deteriorates which leads to more negative thoughts. Patients are lost in their own cognitive distortions, and the negative emotions continue to strengthen, which leads to depression. Specifically, cognitive distortions reinforce negative emotions and thoughts and lead to an overall negative view of the world and a depressed mental state. Cognitive distortions are described as 11 thought patterns, as shown in Table 2. CBT believes that by reversing the user's cognitive distortions, the user's depression can be treated.

**Table 2:** Eleven common cognitive distortions and their descriptions (14).

**Category**	**Description**
Dichotomous reasoning	See things in terms of extremes—something is either fantastic or awful. Believe you are either perfect or a total failure.
Disqualifying the positive	Acknowledge positive experiences but reject them instead of emb racing them.
Emotional reasoning	Believe your emotions are fact
Fortune-telling	Make conclusions and predictions based on little to no evidence and hold them as gospel truth.
Labeling and mislabeling	Assign judgments of value to ourselves or to others based on one instance or experience.
Magnification and minimization	Exaggerate or minimize the meaning, importance, or likelihood of things.
Mental filtering	Focus on a single negative piece of information and excludes all the positive ones.
Mindreading	Inaccurately believe that you know what another person is thinking
Overgeneralizing	This sneaky distortion takes one instance or example and generalizes it to an overall pattern.
Personalization	Take everything personally or assign blame to yourself without any logical reason to believe you are to blame.
Should statements	Make “should” statements.

#### 2.1.2. Cognitive distortion modeling

Cognitive distortions are reflected in the use of language. A set of cognitive distortion words is proposed by previous research, and posts containing these words are cognitive distortion posts (11). And Some research tries to use the Linguistic Inquiry and Word Count (LIWC) dictionary (15), which contains more than 32 categories of psychological catalog and decision tree methods to judge whether there is a cognitive distortion in a person's speech (16).

We argue that cognitive distortions can be learned by natural language models. Current natural language models can detect whether a post contains cognitive distortions. We discuss the cognitive distortion identification task below.

### 2.2. Cognitive distortion classification task

In this section, we discuss how we design and model the cognitive distortion classification task. We use existing natural language models to learn from the dataset we build. After learning, the model can detect cognitive distortions. Later, this method will be used to improve the depression detection model performances.

#### 2.2.1. Cognitive distortion corpus

Leveraging natural language processing techniques requires a dataset. However, to our knowledge, there is no publicly available corpus of cognitive distortion detection. Annotating cognitive distortion labels requires professional knowledge and skills in psychology and it is difficult to annotate for annotators without professional skills. It requires specialized psychology practitioners to consume huge labor and time. To avoid this, we use some data from the internet and then expand to the size we need.

##### 2.2.1.1. Collecting data from the internet

To obtain reliable annotations using the lowest cost, we employ a new data acquisition method. We first construct a small-scale dataset that obtains the data from two sources: We use examples from published research papers on cognitive distortions and examples from articles on the web that introduce cognitive distortions. The distribution of our dataset is shown in Table 3.

**Table 3:** Main statistics of cognitive distortion dataset.

**Category**	**Number**
Dichotomous reasoning	34
Disqualifying the positive	34
Emotional reasoning	25
Fortune-telling	29
Labeling and mislabeling	22
Magnification and minimization	39
Mental filtering	15
Mindreading	34
Overgeneralizing	46
Personalization	44
Should statements	31
All cognitive distortion	353
Normal	1,000

##### 2.2.1.2. Expanding social media data

We have constructed a cognitive distortion dataset based on experts. However, the amount of data in our dataset is still too small for existing deep learning techniques. These data are not generated on the social media platform, so there may have gaps with data from social media. If our data distribution is inconsistent, it will not help social media depression detection.

To obtain a larger dataset of social-media-based cognitive distortions posts, we augment the data on social media in a semi-supervised manner. We train a classifier on our small dataset and then use this classifier to classify social media data. This will generate soft labels. We pick data that have high confidence as our new dataset.

We choose 100 posts randomly and check their labels to make sure their getting correct labels from pre-trained model. As shown in Table 4, we show the precision from the 100 posts we extracted. It can be seen that the precision of the pseudolabels varies significantly on different classes. This is mainly due to the differences in the difficulty of detecting the different classes. Overall, the precision of pseudolabels is acceptable.

**Table 4:** Percision of manual inspection of pseudo labels.

**Catagory**	**Pre.(%)**
Dichotomous reasoning	67.00
Disqualifying the positive	52.00
Fortune-telling	86.00
Emotional reasoning	43.00
Mindreading	78.00
Mental filtering	33.00
Overgeneralizing	91.00
Personalization	56.00
Should statements	98.00

We randomly selected 100 posts and checked the results.

In this way, we expand our dataset and obtain a dataset entirely consisting of social media posts, which makes the use of deep learning technology possible. The data distribution after semi-supervised learning is shown in Table 5.

**Table 5:** Main statistics of cognitive distortion dataset after semi-supervised.

**Category**	**Number**
Dichotomous reasoning	128
Disqualifying the positive	56
Emotional reasoning	77
Fortune-telling	229
Labeling and mislabeling	142
Magnification and minimization	144
Mental filtering	32
Mindreading	233
Overgeneralizing	346
Personalization	84
Should statements	131
All cognitive distortion	1,644
Normal	2,000

#### 2.2.2. Classifying cognitive distortion

BERT, a pre-trained deep learning language model, is used extensively in our research. We use it as the semi-supervised classifier and base model. BERT is an advanced natural language processing model and has been trained extensively on large-scale data. An advanced classifier can be easily obtained by fine-tuning BERT on a small dataset, so we choose it as our base model.

Actually, BERT is a Transformer (17) encoder and already optimized. There are two training tasks for BERT. The first one is to predict masked words and the second one is to determine whether two sentences are related. The researchers believe that in this way, the model can understand the semantics.

**Figure 2:**
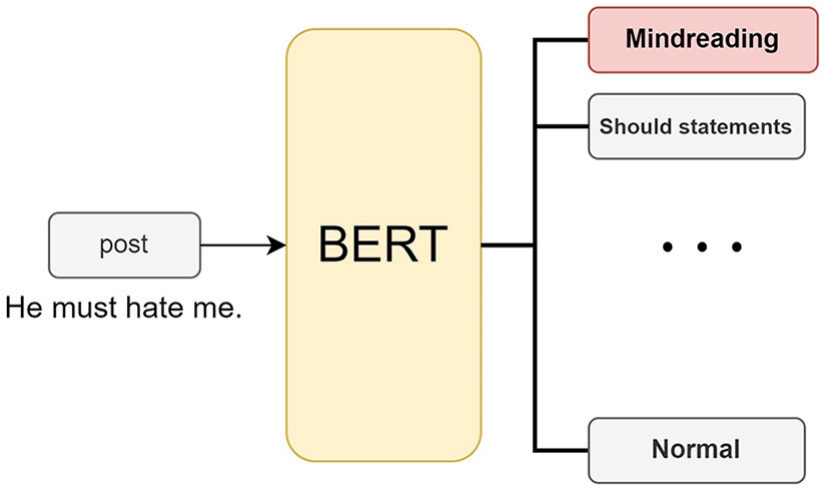
We use BERT to identify cognitive distortions. Users input the text, and the model can output the categories of cognitive distortions it contains.

A cognitive distortion classifier can be trained by learning a cognitive distortion dataset using BERT. We treat cognitive distortion detection as a classification task. As shown in Figure 2, the input is a post on social media, and the output is the type of cognitive distortion contained in the post. The model outputs a total of 12 labels, including 11 cognitive distortions and no cognitive distortions.

### 2.3. Cognitive distortion based explainable depression detection model

According to cognitive distortion theory, cognitive distortion can result in depression. Cognitive distortion detection is closely related to depression detection, and both of them can be expressed through language. We use the cognitive distortion detection task to guide the model for the depression detection task. We believe that modeling cognitive distortions can directly help the model improve depression detection performance. At the same time, cognitive distortion detection will lead to a better explanation of the depression detection model.

We think the model will benefit from combining both tasks. To achieve this, a multi-task architecture is introduced into our model. Multi-task tries to use useful information from multiple closely related learning tasks to help machines model more accurately and is a commonly used modeling method in machine learning. Because of the close relationship between cognitive distortions and depression, we think it would be appropriate to use multitask model to model them.

#### 2.3.1. Model overview

We describe a neural network architecture for performing text classification over multiple input texts. We propose our model based on this architecture for performing two tasks in the social media and mental health domains.

**Figure 3:**
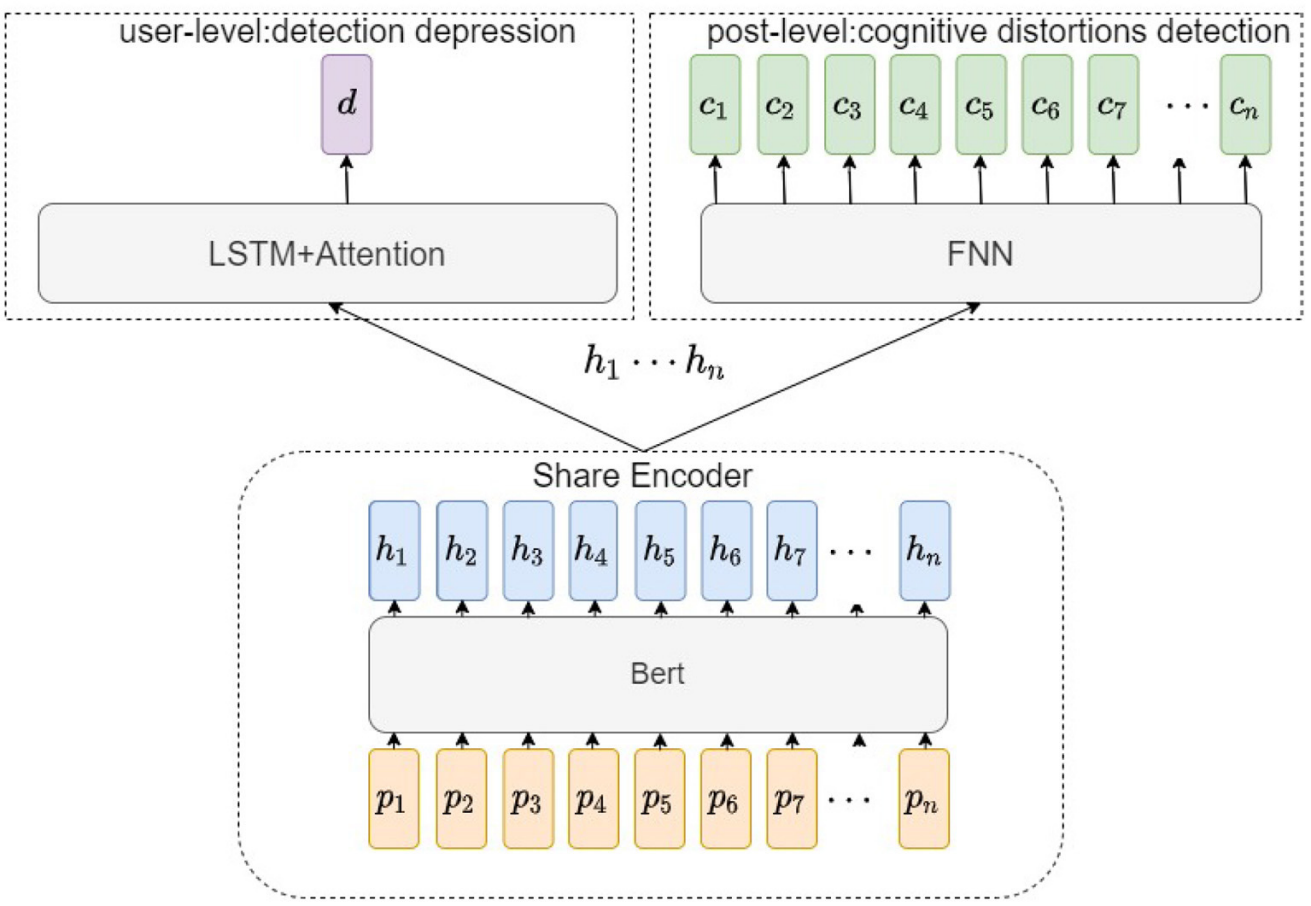
This is our model's structure. Our model uses user posts as input and produces two outputs, cognitive distortion predictions for posts vs. depression predictions for users.

We use the pre-trained technique and multi-task on the architectures of our model. Figure 3 summarizes the flowchart for the screening process based on our models. The inputs of our models are the users' posts. There are two kinds of output, cognitive distortion predictions for each post and depression predictions for each user. At the user-level, the model needs to decide whether the user suffers from depression. At the post-level, the model needs to output the probability distributions of the eleven classes of cognitive distortion. Use the useful information contained in multiple learning tasks to help learn a more accurate learner. In our architecture, there are two tasks at different scales. The main task is the detection of user-level depression. Whereas, cognitive distortion recognition works at the post level as an auxiliary task. Our multitasking arrangement is such that aggregation is done at the user level after explicitly training the cognitively distorted information within the post.

#### 2.3.2. Post-level cognitive distortion detection

This part is completed with the data obtained by the semi-supervised classifier and does not involve data aggregation. At post-level, we directly map the encoded data to the cognitive distortion category. The pretrained model has a powerful learning ability to learn the cognitively distorted language.

In this module, all posts will be fed into the encoder to get the text representation of posts. This encoder is shared between the two tasks. User-level issues are not involved here, so $h_{t}^{i}$ is abbreviated as *h*_*t*_ in this. The process formula is as follows:

(1) $$\begin{array}{l} \left\{h_{1},\ldots ,h_{t},\ldots ,h_{n}\right\}=Encoder\left(\left\{p_{1},\ldots ,p_{t},\ldots ,p_{n}\right\}\right) \end{array}$$

It is then mapped to 12 classification results through a Feedforward Neural Network (FFN).

(2) $$\begin{array}{l} \left\{c_{1},\ldots ,c_{t},\ldots ,c_{n}\right\}=FFN\left(\left\{h_{1},\ldots ,h_{t},\ldots ,h_{n}\right\}\right) \end{array}$$

*p* represents the user's posts, and *h* represents the hidden representation. The *Encoder* is BERT in our implementation. *n* is the number of input posts. *c* is the prediction result of the model. ĉ is the real label for cognitive distortion.

We use the cross-entropy loss function. The machine learning process guides the model to make correct predictions by optimizing the loss function. In the loss function, ĉ_*t*_ is the true cognitive distortion label for the i-th post. *c*_*t*_ is the predicted label given by the model.

(3) $$\begin{array}{l} l_{1}=-\frac{1}{n}\overset{n}{\underset{t=1}{\sum }}{ĉ}_{t}\cdot log\left(c_{t}\right) \end{array}$$

Through this auxiliary task, BERT's encoding results will contain information related to cognitive distortions. This information is helpful for the next step in the model to detect depression. At the same time, the prediction results can also be displayed to professionals to further understand the user's psychological state.

#### 2.3.3. User-level depression detection

For user-level depression detection, it is necessary to combine all the user's posting history information. We have added information about cognitively distorting judgments to each post through multi-task learning. After encoding, we use Long short-term memory (LSTM) (18) Attention (19) to aggregate information. LSTM can get contextual information for each post and aggregate information from user posts. User posts contain a lot of useless information, and Attention automatically filters and aggregates relevant posts. Finally, we get our depression prediction results through FFN.

(4) $$\begin{array}{l} \left\{{ĥ}_{1}^{i},{ĥ}_{2}^{i},\cdots \;,{ĥ}_{n}^{i}\right\}=LSTM\left(\left\{h_{1}^{i},h_{t}^{i},\cdots \;,h_{n}^{i}\right\}\right) \end{array}$$

(5) $$\begin{array}{l} {ĥ}_{all}^{i}=Attention\left(\left\{h_{1}^{i},h_{t}^{i},\cdots \;,h_{n}^{i}\right\}\right) \end{array}$$

(6) $$\begin{array}{l} {\hat{d}}^{i}=FFN\left({ĥ}_{all}^{i}\right) \end{array}$$

Symbols have the same meaning as above. *d* is the model predicting depression outcome. We use the cross entropy loss function. *N* is the number of users entered.

(7) $$\begin{array}{l} l_{2}=-\overset{N}{\underset{i=1}{\sum }}{\hat{d}}^{i}\cdot log\left(d^{i}\right) \end{array}$$

In this part, the model aggregates posts with cognitively distorted information to get the final result. The model can improve its own depression detection performance using detecting cognitive distortion as an auxiliary task.

During training, we optimize two training objectives simultaneously, the model needs to detect both cognitive distortions and depression.

The final loss function is:

(8) $$\begin{array}{l} l=l_{1}+l_{2} \end{array}$$

## 3. Experimental result

### 3.1. Metrics

We use specificity, precision, and F1-score commonly used in the computer field to evaluate the performance of the model. The calculation of these indicators is as follows:

*TP* = The user is a depressed user and the model predicts correctly.

*TN* = The user is a non-depressed user and the model predicts correctly.

*FP* = The user is a depressed user and the model predicts wrong.

*FN* = The user is a non-depressed user and the model predicts wrong.

(9) $$\begin{array}{l} specificity=\frac{TP}{TP\;+\;FN} \end{array}$$

(10) $$\begin{array}{l} precision=\frac{TP}{TP\;+\;TN} \end{array}$$

(11) $$\begin{array}{l} F1-score=\frac{2}{\frac{1}{specificity}\;+\;\frac{1}{precision}} \end{array}$$

### 3.2. Depression corpus

For social media datasets, we use two public datasets, the eRisk-2018 (20) and Clpsych-2015 datasets (21). The annotation process of these datasets is divided into two steps. They use a web crawler to retrieve posts similar to: "I suffered from depression" or "I diagnosed with depression" on social media platforms. Then they let professionals judge whether the post is credible or just a joke. If it is a credible description rather than a joke, they will collect the user's posting history.

The datasets are annotated in this way by automatic and manual work. It ensures a wide range of data sources and also guarantees the authenticity and reliability of the data.

Although these data are all collected on social media platforms, they are collected on different kinds of social media platforms. The distribution of the data is shown in Table 6.

**Table 6:** Main statistics of the train and test collections.

**Data-set**		**Train**		**Test**	
		**Depression**	**Control**	**Depression**	**Control**
eRisk-2018	Num.subjects	135	752	79	741
	Num.posts	49,557	481,837	40,665	504,523
	Avg num. of posts per subject	367.1	640.7	514.7	680.9
	Avg length.of per posts	45.13	34.11	45.38	39.07
CLPsych-2015	Num.subjects	327	572	150	300
	Num.posts	742,793	1,250,606	389,477	727,538
	Avg num. of posts per subject	2,271.5	2,193.4	2,596.5	2,425.1
	Avg length.of per posts	13.91	13.52	13.88	14.00

Among them, the Clpsych-2015 dataset contains more detailed user-level features, which allows us to do some additional analysis.

### 3.3. Result

We test our model on two datasets. Our result shows that performing cognitive distortion detection as an auxiliary task effectively improves the sensitivity and specificity of our model. We compared outcomes with and without cognitive distortions as auxiliary tasks. Our result as the Table 7 shows:

**Table 7:** *F*1. represents F1 score, *Sep*. represents specificity, and *Pre*. represents precision.

**Dataset**	**Model**	***F*1.(*%*)**	***Sep*.(*%*)**	***Pre*.(*%*)**
CLPsych2015	**Bert(multitask)**	**77.49**	**78.68**	**76.60**
	Bert	76.19	77.00	76.37
eRisk2018	**Bert(multitask)**	**64.47**	**64.72**	**64.18**
	Bert	62.70	64.18	60.36

And the method we use and the values obtain have been bolded.

Our model achieves improvements in all metrics. This proves the correlation of the two tasks from a computing perspective, and multi-task learning only improves the model's performance when the two tasks are related. We believe that the identification of mental illness can be improved in the future by adding psychological auxiliary tasks. In the future, depression detection can also be enhanced from the perspective of improving cognitive distortion recognition.

## 4. Discussion

### 4.1. The prevalence of cognitively distorted language in depression people

Some previous researchers have proved the relationship between cognitive distortion and depression via questionnaires (22). However, the questionnaires cannot track people's daily life and are disturbed by the state of the respondents who fill in the questionnaires.

In this section, we will demonstrate that depression patients express more cognitive distortions in their post histories than normal users via natural language processing technology. We explore the association of cognitive distortions with depression users' posts on the Clpsych-2015 dataset.

We use cognitive distortions as a percentage of all posts on behalf of the prevalence of user cognitive distortions. We define the prevalence as follows:

(12) $$\begin{array}{l} p_{popularity}=\frac{N_{cogn}}{N_{normal}\;+\;N_{cogn}} \end{array}$$

Where *N*_*normal*_ is the number of normal post data and *N*_*cogn*_ is the number of posts with cognitive distortions.

**Figure 4:**
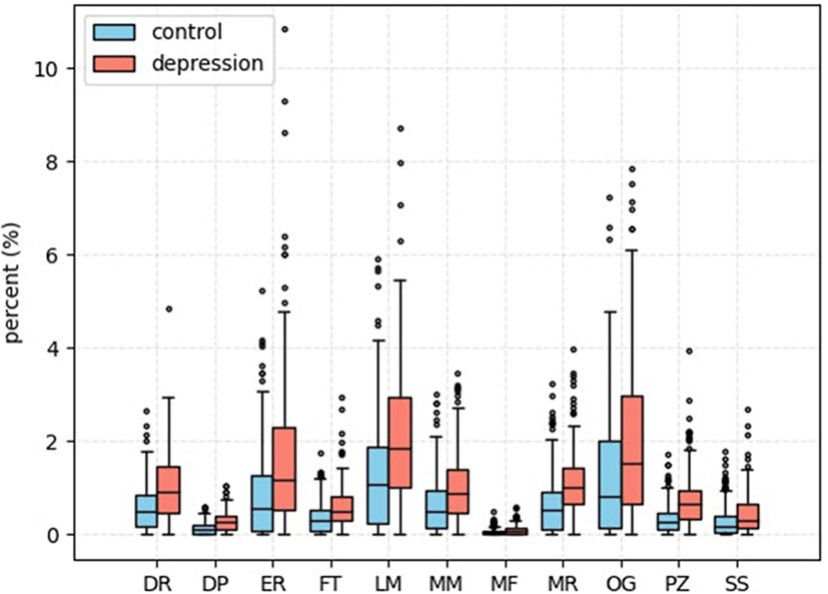
This is a boxplot of various cognitive distortions. The vertical axis is the percentage of total information, and the horizontal axis is the abbreviation for the type of cognitive distortion.

We use deep learning technology to prove that depression patients have higher levels of various cognitive distortions than normal users and validate psychological theory about the relationship between cognitive distortions and depression. The results are shown in Figure 4.

In Figure 4, we can also find some differences in the model's detection result of various cognitive distortions. The model has the worst detection ability for mental filtering. The model only detects a small number of posts that contain mental filtering content. We believe that is because of the imbalance of the data when we collect it. And the characteristics of social media platforms themselves are also related to this.

For example, mental filtering is defined as users ignoring the positive parts of the event and focusing on the negative aspects of the event. This is difficult to reflect on social media posts. However, labeling and mislabeling, defined as labeling oneself or others has become a more common cognitive distortion when users interact with each other on social media.

### 4.2. Cognitive distortion in adolescents with depression

In this section, we show how cognitive distortions change with age, including changes in frequency and content. This research will provide inspiration for how to treat depression patients of different ages, especially adolescents.

As far as we know, this analysis is the first to analyze cognitive distortions based on social media among different ages. Before our research, the usual cognitive distortion data must be obtained through the psychologist in the interview to understand the cognitive distortion information. The problem is that none of these analyzes can be used as a real experience of everyday life, and the amount of data is few.

#### 4.2.1. The frequency of cognitive distortions in different age groups

**Figure 5:**
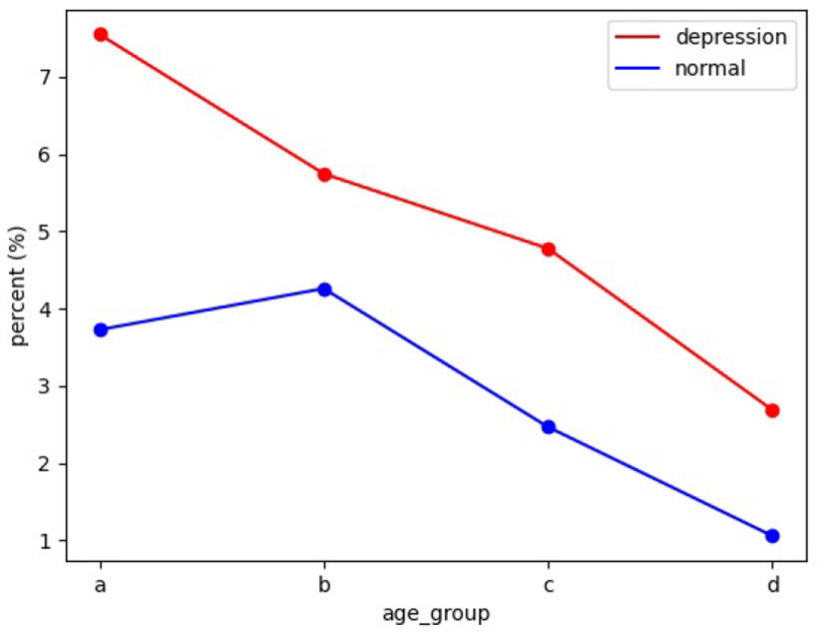
This figure shows the change in the number of tweets with cognitive distortions on social media platforms with age. The horizontal axis is the group, and the vertical axis is the percentage of total cognitively distorted tweets.

We try to analyze the differences in the frequency of cognitive distortions on social media between people with depression and normal people in different age groups. We still use the above *p*_*popular*_ to describe the frequency of cognitive distortions. We divide data into a, b, c, and d four age groups, indicating early adolescence (10–15), late adolescence (16–22), adolescence (22–35), early middle-aged (22–35), and middle-aged (35-). Our first two age groups are based on secondary school and university levels. We define the last two age groups as post-college until age 35 when most people have just graduated and started working, and after age 35, when most people enter a stable life stage. We calculate the average frequency of occurrence of cognitive distortions across age groups. The results of our analysis are shown in Figure 5.

We find that cognitive distortions detected on social media tended to decline broadly with age, in both depressed and normal individuals. We think there are two reasons for this: Adolescents are at a time when their perception of the world is forming. This age group involves a profound amount of change in all domains of development–biological, cognitive, psycho-social, and emotional. It is easy to create unreal perceptions of the world, lives, and themselves. At the same time, we cannot deny that people of different ages express themselves differently on social media. Adolescents prefer to express themselves and share their lives on social media, while other age groups are more likely to use it as a connection tool, so the data obtained from social media may have bias.

#### 4.2.2. The content of cognitive distortions in different age groups

**Figure 6:**
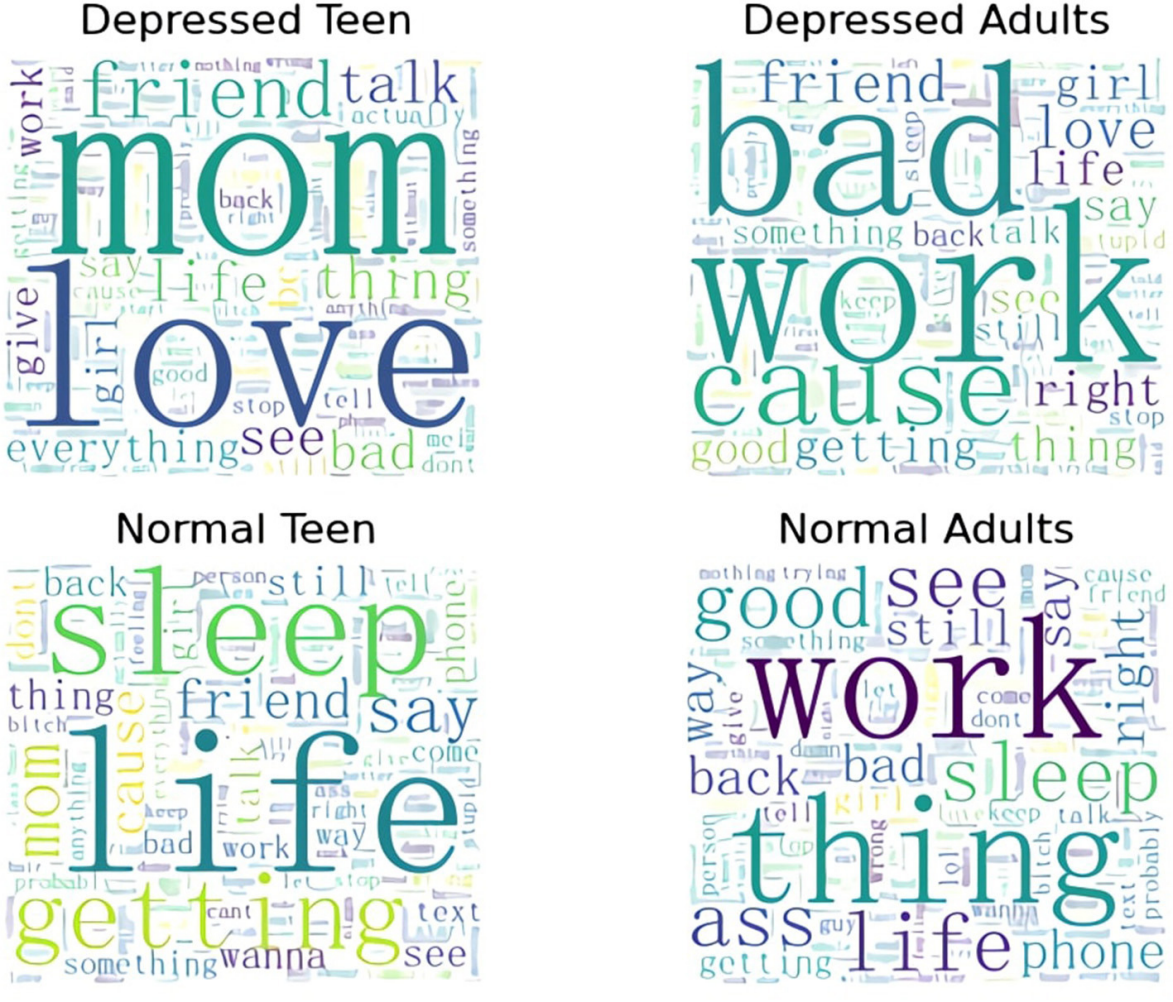
This image shows which words previous cognitive distortions focus on in various groups of people.

We count word frequencies in cognitively distorted sentences in data across age groups and produced word clouds. As shown in Figure 6, there are some differences between different age groups:

For adults with depression, their cognitive distortions come from work stress and a more trivial life. Their cognitive distortions focus on work.

For adolescents with depression, **“mom,” “friend,”** and **“love”** are the most frequently mentioned by adolescent depression patients. For the increased frequency for **“mom,”** We think that the role of the mother in the family is the key role in producing cognitive distortions in adolescents with depression. This may be because traditionally mothers have more parenting responsibilities and time than fathers. The importance of mothers is much higher than other family members. We think that the presence of **“friends”** represents social problems in adolescents with depression. As cognitive distortion theory points out, cognitive distortions reinforce social withdrawal, and social withdrawal continues to reinforce cognitive distortions. At last, the high frequency of **“love”** indicates that lack of love in life is also one of the factors that result in depression.

For normal people, the positive vocabulary in the language increases a bit. This also shows that the cognitive distortion we are talking about is not a negative emotion, but a thinking pattern.

### 4.3. Case study

We present here the output of our model to show how the model can assist professionals in CBT. We will show the cognitive distortion languages extracted by the model, and the distortions present in these sentences. Psychologists can treat people with depression by reversing the distortions.

**Figure 7:**
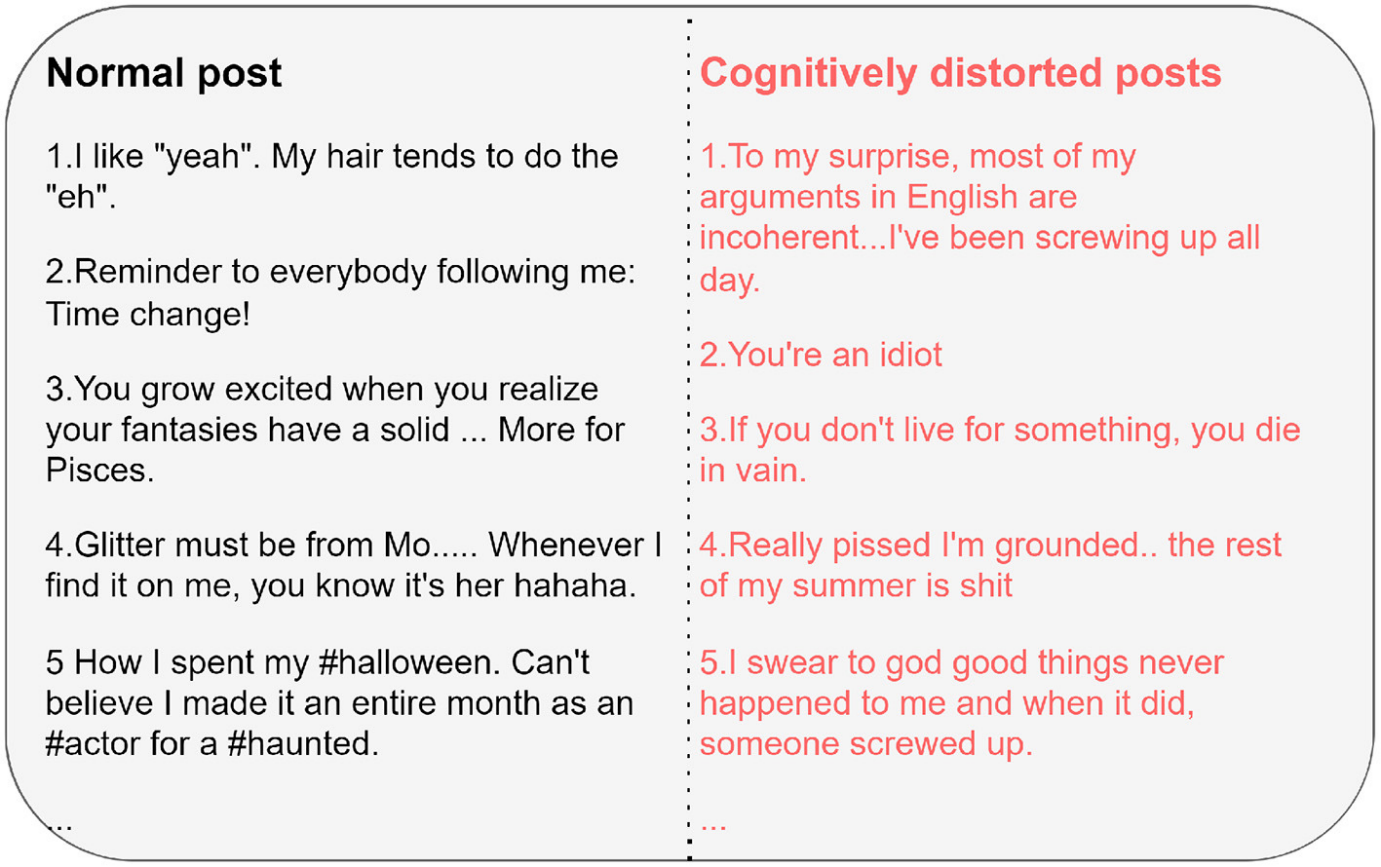
This figure shows the difference between cognitively distorted extracted text and regular text. Professionals can use the text processed by the model to examine the psychological situation of users and treat them.

Our system gives cognitive distortion language after detecting people with depression. We see a large number of cognitive distortion languages in depression patients. As shown in Figure 7, the language posted by the user has no direct explicit connection with depression and is easily ignored by the model. But psychologists can see that users have cognitive distortions in their perception of reality, which is an important signal of depression. With our approach to modeling cognitive distortions, models can capture this difference.

In the process of CBT for depression must first find out the cognitive distortions of users, which are often obtained through the analysis of user conversations. And depression patients get many cognitive distortions in their everyday lives. After identifying a depressed patient and discovering his cognitive distortions, the next step for professionals is to reverse them. We leave both steps to the computer to complete, which saves a lot of time and allows psychologists to help patients recover as quickly as possible.

### 4.4. Conclusion and future work

In our study, we construct a system that can perform both tasks of depression detection and cognitive distortion detection simultaneously, combining two psychologically closely related tasks and making them work together to improve depression recognition reliability and accuracy. We the first attempt to study cognitive distortions in people with depression using deep learning technology. At the same time, we analyze the output of the model, especially the cognitive distortion phenomenon in depressed adolescents, and obtained several key factors affecting the cognition of adolescents with depression. Technically, we build a cognitive distortion corpus and a multi-task learning model and combine psychological theory with natural language processing technology. From a theoretical point of view, we have verified the psychological conclusions and drawn some new psychological findings based on natural language processing technology.

In the future, we believe that the combination of computing technique, especially artificial intelligence research and psychology is inevitable. We believe that automated models will help psychologists better analyze data, and psychological theory can also feed the development of automated models. We hope to explore how cognitive distortions can be better calculated from a computer's point of view. And hope for more reliable, better-understood algorithms that automatically identify depression.

## Data availability statement

The raw data supporting the conclusions of this article will be made available by the authors, without undue reservation.

## Author contributions

BW and YZ conceived the idea of this study and collected the necessary data for this study. BW and XL jointly designed the experimental procedure for this study. BW developed the tools required for the experiments, performed the entire experimental procedure, and wrote the manuscript with input from all authors. BQ and XL give important references. All authors contributed to the article and approved the submitted version.
